# An Oxygen Supply Is Not Enough: A Qualitative Analysis of a Pressure Swing Adsorption Oxygen Plant Program in Ethiopian Hospitals

**DOI:** 10.9745/GHSP-D-23-00515

**Published:** 2024-08-27

**Authors:** Victoria Smith, Alana Changoor, Sarah Rummage, Haileab Fekadu Wolde, Ejigu Gebeye Zeleke, Getahun Mekonnen Belay, David Barash, James Stunkel, Cheri Reynolds

**Affiliations:** a Assist International, Ripon, CA, USA.; b Grand Challenges Canada, Toronto, Canada.; c Department of Epidemiology and Biostatistics, Institute of Public Health, College of Medicine and Health Sciences, University of Gondar, Gondar, Ethiopia.; d Faculty of Health Sciences, Curtin University, Bentley, Australia.; e Telethon Kids Institute, Nedlands, Australia.; f Assist International, Addis Ababa, Ethiopia.; g GE Foundation, Boston, MA, USA.

## Abstract

**Background:**

In response to critical gaps in medical oxygen access, 2 pressure swing adsorption (PSA) oxygen production centers were established using an ecosystem-strengthening strategy in Amhara, Ethiopia, in 2019. A qualitative study was conducted to assess enablers and bottlenecks to oxygen access at the hospital level after installation.

**Methods:**

A variety of hospital staff (clinicians, biomedical professionals, hospital administrators, and procurement teams) across 13 hospitals procuring oxygen from the plants participated in comprehensive, semistructured focus group discussions. A thematic framework analysis approach was used to identify key themes.

**Findings:**

A total of 101 individuals participated in 26 focus groups in 2021, 2 years after plants were installed. Primary themes were accessibility of supply, affordability, and hospital readiness. Respondents indicated a substantial increase in their hospital’s ability to access lower-cost oxygen, with many attributing this to the locality of plants and reduced transportation barriers. However, other challenges persisted, and the emergence of COVID-19 1 year after plant installation and a civil conflict exacerbated supply shortages. Investments in equipment, supplies, and training optimized clinical utilization of oxygen and were highlighted as a need for ongoing investment.

**Conclusion:**

To achieve maximum impact, investments in large-scale oxygen systems must be accompanied by strategic plans to transport oxygen, reduce costs to hospitals, and provide support to clinical teams through equipment, supply procurement, and clinical training. These findings support comprehensive ecosystem approaches to strengthening oxygen access for sustainable impact.

## Abstract (precis)

Pressure swing adsorption (PSA) oxygen systems are more complicated than oxygen concentrators but can generate a much greater volume of medical oxygen and serve a network of hospitals, increasing regional supply. Direct feedback from hospital workers collected during the COVID-19 pandemic provided strong validation and reinforcement of the need for new oxygen supplies to be accompanied by investments in transportation, clinical and technical training, and provision of equipment and supplies.

## Teaser

**Key Findings:**

- Locating large-scale pressure swing adsorption (PSA) oxygen plants near high-volume specialized hospitals had a transformational effect, increasing the supply of locally available oxygen.
- Transportation and distribution are key barriers in large-scale oxygen production systems like PSA plants or liquid oxygen plants and can make up a large percentage of total oxygen costs to hospitals. Reducing transportation distance can dramatically reduce oxygen costs. However, even short-distance distribution can be limited by the capacity and availability of hospital vehicles to pick up oxygen. Planning for short-distance distribution is key to reducing oxygen shortages and stock-outs in hospitals that rely on cylinders.

**Key Implications:**

- Policymakers should account for the geographic distribution of oxygen supplies and factor transportation methods and costs into long-term national oxygen planning if not all hospitals or health centers will have their own means of oxygen production on site.
- To achieve maximum efficacy, oxygen supply sources like concentrators, liquid oxygen, and PSA plants must be paired with supplies like oxygen consumables, equipment like pulse oximeters, and clinical training.

## INTRODUCTION

Medical oxygen is a foundational element of care for a variety of health conditions, required for approximately 20% of hospitalized neonates, 13% of hospitalized pediatric patients,^1^ certain obstetric emergencies, medical and trauma-related conditions, and safe surgery.^2^^,^^3^ The COVID-19 pandemic resulted in surges of critically ill patients who needed large oxygen volumes, taxing already stretched health systems.^4^^–^^6^ While efforts to quantify the oxygen access gap are ongoing, current evidence suggests substantial shortfalls that predominantly affect low- and middle-income countries.^2^^,^^7^^,^^8^ For example, a recent study of 64 intensive care units across 10 African countries found that 1 in 2 patients with COVID-19 died without receiving medical oxygen.^9^

Before the COVID-19 pandemic, large-scale programs to improve access to medical oxygen in resource-constrained settings were relatively rare.^10^^,^^11^ Most interventions evaluated in the literature deployed oxygen concentrators,^12^^–^^16^ and many achieved successful reductions in mortality.^12^^,^^13^^,^^17^ These programs generated key learnings about the ecosystem of interventions needed, including clinical trainings, pulse oximeters, and support for local biomedical teams.^16^^,^^18^ Despite the existence of several mixed-method studies on oxygen concentrator systems at the time of writing,^7^^,^^13^^,^^19^^–^^24^ interventions that provide larger-scale oxygen production volumes (e.g., liquid oxygen [LOX] and pressure swing adsorption [PSA] plants) are notably absent from the literature, despite presenting different requirements than smaller-scale concentrator programs.^16^^,^^25^

With the emergence of the COVID-19 pandemic, the role of larger-scale oxygen systems has become increasingly relevant because of their ability to provide oxygen supplies across broader geographies and to significantly greater volumes of patients.^25^^,^^26^ For example, as of December 2022, a total of 267 PSA oxygen plants were being procured or repaired by large organizations, such as Unitaid, the Global Fund, and UNICEF, as part of the World Health Organization’s Access to COVID-19 Tools Accelerator.^27^ A dearth of implementation research on the determinants of success for PSA systems represents a key gap in the literature.

In early 2019 (before the COVID-19 pandemic), Assist International partnered with the GE Foundation, Grand Challenges Canada, and the Amhara Regional Health Bureau (ARHB) to expand regional oxygen availability by implementing a PSA oxygen demonstration program in Amhara, Ethiopia. Two PSA plants based at referral hospitals supplied medical oxygen to the host hospitals and other hospitals in the region. This program adopted an ecosystem approach that included the provision of clinical training, equipment, and supplies at 22 program hospitals and the development of an innovative business model to reduce costs. Although some ecosystem approach definitions may also include changes in policies, norms, and standards, the scope of this article was limited to hospital-level influence with components also defined in our previous research.^25^ Our findings indicated that each plant has been directly providing life-saving medical oxygen to over 22,000 patients per year at a cost of US$7.34 per patient treated.^25^ The current study builds on previous research by capturing direct hospital feedback on the oxygen program during the COVID-19 pandemic. The goal of this research was to clarify the determinants of success and bottlenecks to impact for PSA plants. These findings can be used to drive evidence-based decision-making and resource allocation needed to optimize the utility and longevity of new oxygen investments.

This research aimed to clarify the determinants of success and bottlenecks to impact for PSA plants.

## PRESSURE SWING ADSORPTION OXYGEN DEMONSTRATION PROGRAM

This study was designed to evaluate the impact of a PSA oxygen demonstration program that included 2 large-scale PSA plants (installed in 2019) in Amhara, Ethiopia (Figure). The location of the PSA plants was determined according to Ethiopia’s national oxygen roadmap, which outlined the Ministry of Health’s priorities. In addition to the 2 plants, the program also invested in a business component for financial sustainability, clinical and technical training, regular maintenance, and accessories and consumables as part of its ecosystem approach. The program developed a public-private partnership with the ARHB, with plants at regional specialized hospitals serving as localized oxygen supply hubs for the surrounding spoke hospitals that were located between 12 km and 180 km away from the hub hospitals, with the average distance just over 100 km (Table 1). Some hospitals were small and located in remote mountain geographies with poor road access, meaning that despite comparatively short distances, travel times to any oxygen source were significant. Before the plants were installed, the average distance to an oxygen source was about 200 km, with some hospitals reporting oxygen sources more than 400 km–500 km away. Completing a round-trip oxygen delivery is feasible in a single day at a distribution distance of 200 km, depending on the road quality and routes of travel from the plants to hospitals in the Amhara Region. Longer travel times create oxygen stock management challenges because hospital staff must estimate how quickly the inventory of available oxygen will be depleted and how much time is needed to travel to replenish the inventory before supplies are exhausted. Therefore, shortening these routes was an important part of the program design.

**FIGURE:**
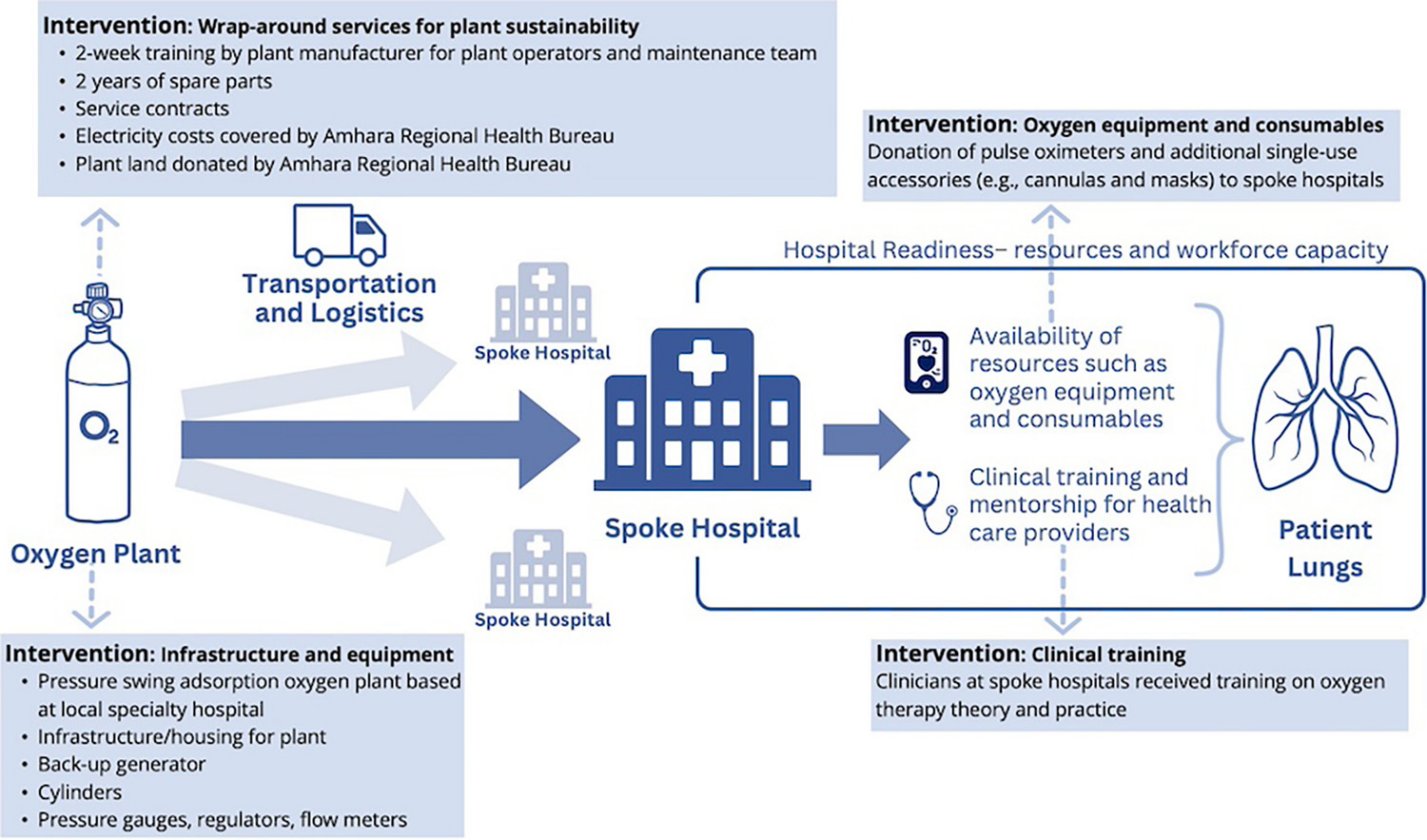
Intervention Components of Pressure Swing Adsorption Oxygen Demonstration Program in Amhara, Ethiopia

**TABLE 1.** Distance to Pressure Swing Adsorption Plant From Spoke Hospitals, Amhara, Ethiopia

**Hospital**	**Distance, km**
1	85
2	140^a^
3	180^a^
4	61
5	35
6	130
7	12
9	100^a^
10	75
11	178
12	120

^a^ Remote and/or mountainous location with challenging roads.

Capable of producing just over 750 cubic meters of oxygen per day or 125 6-cubic meter oxygen cylinders each in 24 hours, the AirSep AS-J duplex plants with booster compressors were established at Bahir Dar and Dessie Specialized Hospitals (with 430 and 311 beds, respectively). Plants were staffed to run 24 hours per day, although they ran less if less demand, electrical outages, or equipment downtime occurred. They were equipped to supply oxygen regionally to surrounding primary and general hospitals, with revenues from low-cost oxygen sales reinvested into the plant for sustainability.

At the time of program implementation, most program hospitals had no piping. The few that did have any piping only had 1 or 2 wards piped through a connection to a manifold, with most oxygen being provided to patients via cylinders next to the patients’ bedsides. Although hub hospitals received PSA oxygen plants on site, wards were not piped, and patients received oxygen via cylinder. One hub hospital added some piping late in the program, but piping was not available at the program’s inception. Spoke hospitals picked up oxygen with their own vehicles from oxygen plants.

The plants and oxygen cylinders were provided through grant funding from Grand Challenges Canada and the GE Foundation, and land, housing, some plant labor costs, and electricity for the plants were donated by the ARHB. Assist International Medical Oxygen PLC partnered with the ARHB through a public-private partnership to create the Amhara National Regional State Oxygen Production and Distribution Centre (the Amhara Oxygen Center). These partners comanaged the oxygen plants until full ownership was transitioned to the ARHB.

Hospitals pay per cylinder of oxygen, and revenues from these oxygen sales are invested back into the program. However, no hospitals, including the hub hospitals, are expected to provide capital or operational expenditures for the Amhara Oxygen Center from their hospital budgets. While some start-up funds were donated from the initial investors to cover operations, the ARHB and AI Medical Oxygen Production PLC split operational costs until the conclusion of the public-private partnership and the exclusive ownership transitioned to the ARHB. This included ongoing maintenance costs for service contracts and spare parts (electricity costs were already covered by the ARHB, and labor costs were split between ARHB and AI Medical Oxygen Production PLC). After a little over a year, plant revenues were more than sufficient to cover all ongoing expenditures; however, ARHB elected to reserve the funds for oxygen scaling opportunities and ongoing maintenance. More information on both capital and operational expenditures is available in supplement materials of our previous research.^25^

As PSA plant longevity is a common barrier in low-resource settings, the AirSep AS-J duplex plants were selected for their durability and reliability. Routine preventive and corrective maintenance were also imperative to keep the plants functional long-term. Biomedical engineers (who were also plant operators) were trained for 2 weeks by plant manufacturers to implement standard operating procedures, provide routine preventive maintenance, and perform basic troubleshooting operations. Assist International provided 4 years of additional support for ongoing advanced corrective maintenance through in-person coaching, remote live video tele-mentoring and training, sourcing spare parts (paid for through plant revenue), and technical support. Training of plant technicians, spare parts, service contracts, tools, and test equipment were all provided as key elements of the program design to ensure operational sustainability.

Over the course of the program, 688 clinicians from 22 surrounding hospitals received clinical training on the safe and effective administration of oxygen therapy. Program hospitals received additional pulse oximeters, regulators, and flow meters, although these donations did not fully relieve preexisting resource gaps. Health facilities within the geographic scope of the 2 plants serve a catchment of over 20 million people.

## METHODS

### Design and Participants

In 2021, 28 focus group discussions (FGDs) were conducted at 14 hospitals (10 primary, 2 general, and 2 specialized) in the Amhara region, Ethiopia. One hospital did not procure oxygen from the plant and was therefore excluded from the analysis, reducing the total number of focus groups to 26 at 13 hospitals. Research sites were selected due to their proximity to the Amhara Oxygen Center and need for medical oxygen. At each facility, 2 FGDs were conducted: 1 with clinical team members and the other with the hospital administrators, biomedical teams, and purchasing/procurement officers. Several of the authors belonged to the organization implementing the intervention. To minimize bias, no members of the group that funded or implemented the oxygen ecosystem program were present during the FGDs, which were facilitated by researchers from the University of Gondar. Of note, the COVID-19 pandemic and the political crisis in Ethiopia were unexpected events that emerged in 2020 and affected oxygen demand and supply into 2021, when the FGDs were eventually held.

### Data Collection Procedures

Each FGD was conducted in Amharic by 2 experienced qualitative researchers (HFW and EG) with Master’s degrees in public health from the University of Gondar using a semistructured interview guide. Informed consent was obtained from all FGD participants. FGDs were audio-recorded and translated into English by the facilitators, who were native Amharic speakers. Any ambiguous translations were clarified by the program team, with meanings verified by the original translators.

### Data Analysis

Interviews were analyzed using a thematic framework approach. This included data familiarization, identification of a thematic framework, indexing/charting, mapping, and interpretation.^28^

Two data analyzers (AC and SR) with backgrounds in public health and social business enterprise developed a codebook by reviewing interviews holistically to individually identify and then align on general themes through collaborative discussion. Data analyzers then used the coding framework to index transcripts independently. Coding of each transcript was compared across analyzers, and inconsistencies were discussed. If the analyzers were unable to arrive at consensus, a senior team member helped resolve the inconsistency. The data were analyzed, coded, and charted on Microsoft Excel to create a summary matrix. The interpretation of the data was discussed between 3 team members, revised by the FGD facilitators HFW and EGZ, and compared against previous programmatic history and literature to build consensus around 3 primary themes and 6 subthemes.

### Ethical Approval

Formal ethical review and written approval were received for the FGD protocols from the internal review board of the Amhara Public Health Institute.

## RESULTS

A total of 101 nurses, doctors, hospital chief executive officers, purchasers, and biomedical technicians participated in 26 focus groups in 13 hospitals (10 primary, 1 general, and 2 specialized) procuring oxygen from the plant. Overall, 3 themes and 6 subthemes emerged:

- Accessibility: supply and demand; transportation–supply implications
- Affordability: payments and financing; transportation–cost implications
- Hospital readiness: resources (oxygen equipment and consumables); workforce capacity (clinical training)

Table 2 summarizes the enablers and bottlenecks to oxygen use.

**TABLE 2.** Bottlenecks and Enablers to Oxygen Access

**Factor**	**Bottleneck**	**Enabler**
Supply	Inadequate supply of oxygen to meet patient needs.	Oxygen plants established in strategic locations increasing the accessibility of supply.
Transportation	Limited availability of vehicles for transportation; long distance to oxygen source.	Adequate number of vehicles and cylinders to transport necessary quantities of oxygen; shorter distances from hospitals to oxygen plants.
Cost	High cost of oxygen procurement for hospitals and, by extension, for patients.	Close proximity of oxygen plants to hospitals decrease transportation costs; Government guidelines provided around oxygen pricing for patients.
Equipment	Inadequate supply of equipment or consumables limiting ability to provide safe and effective oxygen therapy.	Improved access to supply chains and financing for equipment and consumables such as cylinder regulators, flowmeters, nasal cannulas, and pulse oximeters.
Clinical Skills	Clinicians have limited opportunities for training on safe and effective oxygen therapy administration.	Training is offered to refresh clinicians on safe and effective oxygen therapy administration.

### Accessibility

#### Supply and Demand

Participants at all program hospitals mentioned improvements in the oxygen supply after the installation of the oxygen plants—a key success of the program. Participants at many hospitals that perceived improvements in their oxygen supply cited resultant reductions in outward referrals and mortality.

Participants at all program hospitals mentioned improvements in the oxygen supply after the installation of the oxygen plants.

*As the doctor previously mentioned, having the oxygen plant here is very good. In previous times, we lost many children because of lack of oxygen. Since oxygen is critically important for neonates to survive, the oxygen plant saves the lives of many neonates that could die because of the lack of oxygen.* — Newborn intensive care unit clinician

However, 11 of 13 hospitals stated that despite improvements, the existing oxygen supply was still not sufficient to meet their needs. It was also noted that the plants had been stretched to provide oxygen to an increasing number of hospitals seeking new supply sources during the pandemic. At the time of the FGDs, the plant at Bahir Dar was running at 100% capacity, and Dessie fluctuated between 70% and 94% capacity. Plant production varied based on hospital demand, electricity fluctuations, and occasional periods of downtime. Hub hospitals received the majority of the oxygen supply, with the 2 hub hospitals receiving about 54% of the total cubic meters of oxygen distributed. While the program initially planned for each plant to supply 1 large hub specialized hospital, 1 general hospital, and 5 smaller primary hospitals, 60 different health facilities sought oxygen from the plant at Bahir Dar in 2021, with the plant at Dessie providing oxygen to 46 hospitals (though not all concurrently). Because of the increased oxygen demand due to the COVID-19 pandemic and the large number of hospitals seeking oxygen, many hospitals supplemented their oxygen supply with other oxygen providers at a higher cost.

*This oxygen plant [now] serves most of the hospitals in east Amhara… Therefore, it is better to install other plants in the hospitals with high patient flow.* —Medical director

Although the installation of the 2 plants was perceived as having a positive impact, participants at most hospitals wanted more plants installed across the region to continue to increase the local supply.

#### Transportation: Supply Implications

Participants at most hospitals perceived more local and regionally based oxygen plants to be a considerable advantage, with substantially reduced travel time being a transformative positive change, particularly during a period of civil conflict and road closures. Spoke hospitals averaged a distance of just over 100 km from the plant. However, road quality was a major differentiating factor in estimating oxygen transportation time. Remote, mountainous, and gravel roads all slowed the oxygen distribution system. Purchasing document data from hub and spoke hospitals, along with discussions with purchasing departments, indicated that the average travel time to procure oxygen dropped from about 17.6 hours to 6.4 hours. Despite marked reductions in travel time from previous oxygen suppliers, oxygen resupply time commitments remained a burden for more remote hospitals.

Some participants reported that they would have been completely cut off from oxygen if the plants had not existed, making local oxygen sources critical to enabling certain medical services to continue.

*Now, the Abay Dam is closed because of the instability so it is difficult to get oxygen from Addis Ababa. So, what we could do if we don’t have the Amhara oxygen center at Bahir Dar?* —Service quality head

However, in the absence of a centralized delivery system, hospital staff also stated that their hospital’s limited transportation capacity was a key bottleneck and that they needed more cylinders and vehicles to transport oxygen, a difficulty exacerbated by long wait times at plants.

*Because we get only 6 cylinders in 1 round and we use these cylinders for 6 patients, although there are more than 6 patients who need oxygen in the hospital. Hence, the oxygen supply we have and the number of patients in our hospital are not totally proportional. This is our biggest problem! The main reason for getting a small number of cylinders per round of transportation is the lack of a large vehicle to transport a higher number of cylinders in a single round. Hence, if we can get a large vehicle with better capacity and a high number of cylinders, this problem can be solved*. —Biomedical engineer

### Affordability

#### Payments and Financing

At the Amhara Oxygen Center, hospitals paid an average of Ethiopian Birr (ETB) 282 (∼US$6.46 in June 2021) per cylinder of oxygen (6–8 cubic meters of oxygen per cylinder, size J or K) and covered their own transportation expenses for the oxygen. No hospitals, including the hub hospitals, were expected to cover any plant sustainability costs through the program outside of the cost per cylinder of oxygen. Participants at the majority of hospitals indicated that the cost of oxygen to hospitals had decreased since the Amhara Oxygen Center had opened, with rough purchasing data indicating that the cost at baseline was ETB 439 per cylinder (∼US$15.81 in May 2018 when baseline data were collected, although earlier needs assessments in 2017 were ∼US$21 per cylinder) (same sizes). Some hospitals provided oxygen for free, while others charged patients for treatment, often with exemptions for pregnant women and children. Without charging patients, hospitals reported facing financial losses in covering these costs, and most staff expressed needing clearer guidelines from the government on pricing oxygen for patients.

Hospitals that charged patients reported passing savings from the Center’s lower-cost oxygen to patients, with some hospitals anecdotally estimating a 30%–50% reduction in cost resulting from lower prices and reduced transportation. This anecdotal evidence aligned with purchasing data estimates, which showed a 56% reduction in price.

*We used to buy from Addis Ababa so the cost was expensive. But now the cost of oxygen is reduced by 50%. So, the community is getting the treatment in the nearby hospital, meaning financial losses are reduced. For example, when [before] they had been referred to [Hospital 10], now the transport and food costs are reduced. Therefore, the communities around [Hospital 12] are getting oxygen therapy at [Hospital 12]. By implication, they are getting better service with less cost.* —Chief executive officer

As oxygen procured from the Center was unlikely to fulfill a hospital’s entire need, most hospitals supplemented their supply from private suppliers at a higher cost, creating additional financial strain on hospitals. To mitigate this problem, many hospitals identified a need for government subsidization at the plant level.

#### Transportation: Cost Implications

Having the plants available locally meant transportation costs were significantly less than procuring oxygen from the capital city. The average number of hours from a hospital to the oxygen source dropped from 17.6 hours to 6.4 hours. This conferred substantial cost savings, as transport costs included vehicle rental, fuel, and per diems for traveling staff in their total oxygen costs. Despite significant reductions, costs remained a challenge.

Having the plants available locally meant transportation costs were significantly less than procuring oxygen from the capital city.

*We are transporting the oxygen with our own car. Our [car] can hold only 6 cylinders so we should send the car to the plant every day or every other day. This problem increases the cost that we should pay for fuel and daily allowance of the purchasing staff. Besides, when the car is busy with other hospital activities, we rent a car from private owners with a high cost. Since we are not requesting payment for oxygen therapy, all these costs are causing financial damage to the hospital.* —Finance head

Besides the cost, participants also noted that some hospitals used ambulances to transport oxygen when other vehicles were not available, a practice with potentially serious repercussions, including reduced accessibility of ambulances to transfer sick patients.

### Hospital Readiness

#### Resources: Oxygen Equipment and Consumables

Participants reported that one-time donations of regulators, flow meters, and pulse oximeters were extremely helpful, especially during the COVID-19 pandemic, although the program did not provide the full quantity that the hospitals needed to fully close preexisting resource gaps.

A need for additional pulse oximeters, in particular, was a ubiquitous finding, with many respondents reporting regular shortages in pediatric sensors and issues with hospitals typically procuring low-quality oximeters that broke easily and provided unreliable readings. Lack of key accessories was also a significant impediment to providing patients with oxygen therapy, even if an oxygen supply was available. Respondents reported that they needed more pressure gauges to administer oxygen, and staff at many hospitals reported that a number of gauges they had were broken or did not fit properly, leading to oxygen leakages. Only 3 of 13 assessed hospitals did not report accessories (gauges, flow meters, regulators) as an urgent need.

*Although we have the oxygen, sometimes we are not able to give the oxygen to patients because of a lack of gauges.* —Newborn intensive care unit nurse

Oxygen consumables, such as nasal cannulas and face masks, were single-use supplies that had to be continuously procured. Respondents reported these being in short supply, with a majority of hospitals lacking pediatric and neonatal sizes. Respondents also noted key supplies like accessories and consumables were not always available in local markets and indicated that neonatal consumables were particularly difficult to source.

#### Workforce Capacity: Clinical Training

Clinical training through the program led to reported increases in adherence to best practices, including identifying hypoxemic patients, determining proper flow rates, handling equipment, and reducing oxygen wastage. Respondents from 85% of hospitals perceived themselves as providing better patient care after the oxygen training, and most suggested that the training be offered to more clinicians at their hospital.

*Before the training, I even fear to touch cylinders because I didn’t have any knowledge about oxygen administration and I had skill gaps. However, all this knowledge and skill gaps are solved after I received the training.* —Operating room nurse

Although theoretical content was well covered, several hospitals reported needing additional hands-on practice and refresher trainings.

## DISCUSSION

In the wake of the COVID-19 pandemic, there is growing interest in PSA and LOX oxygen systems as a strategy to significantly boost oxygen supplies to support pandemic recovery and build health system resilience.^27^^,^^29^ To our knowledge, this is the first qualitative assessment of a PSA-based oxygen program investigating enablers and barriers to improved oxygen access.

Overall, this study found that creating a local oxygen supply source at referral hospitals improved the accessibility and affordability of medical oxygen by increasing the volume of oxygen available and reducing transportation barriers. Respondents attributed reductions in outward referrals and mortality to increased accessibility of oxygen. Investment in oxygen supplies like pressure gauges, regulators, flow meters, pulse oximeters, and single-use cannulas and masks, as well as health worker training, were also reported as enablers of optimal oxygen usage.

Overall, this study found that creating a local oxygen supply source at referral hospitals improved the accessibility and affordability of medical oxygen by increasing the volume of oxygen available and reducing transportation barriers.

Nonetheless, the program did not fully resolve the challenge of scarcity. The COVID-19 pandemic greatly amplified oxygen demand, stretching supplies thin. Many hospitals still faced barriers of insufficient cylinder and vehicle capacity to transport oxygen. Moreover, hospitals cited lack of government subsidies as financial barriers to sustainably financing their procurement of oxygen supplies.

A key value-add of the program was its focus on an ecosystem approach. A growing body of evidence indicates that ecosystem approaches are needed to translate increased supplies of oxygen into improved health outcomes.^6^^,^^7^^,^^12^^,^^13^^,^^15^^,^^16^^,^^18^^,^^19^^,^^30^^,^^31^ As several studies have illustrated, some hospitals do not use enough oxygen, even when the volume of available oxygen has increased.^22^ A 2021 systematic review found bottlenecks to oxygen utilization are cross-cutting, including everything from inadequate maintenance, availability of accessories and supplies, and gaps in clinical knowledge and skills.^11^ Before our research was conducted, most evidence on ecosystem approaches had been centered around concentrator-based systems, including projects in Nigeria^14^ and Papua New Guinea,^13^ where provision of oxygen concentrators was supplemented with pulse oximeters, training for biomedical technicians on concentrator maintenance, and clinical training. However, ecosystem considerations for PSA plants are different, and the enablers and barriers to access for these systems warrant additional study.

For example, one of the most significant advantages of PSA-based systems is the volume of medical oxygen they produce, which can serve a network of hospitals. In sizing oxygen plants, the program took into account availability of start-up capital, oxygen demand at the hub facility, lack of piping (piping tends to increase oxygen usage), geographic proximity and density of surrounding health facilities, oxygen demand at target spoke health facilities, electricity costs, sustainability costs, and travel time to oxygen sources. Because of the program team’s experience with similar models in Kenya and Rwanda, the team learned it can take time to build a reliable customer base with a cash flow that can sustainably support operations. There are several key risks in sizing. If the procured oxygen plant is too large, the electricity and running costs to support operations will be very high, making ongoing financial and operational sustainability a challenge. If the plant is too small, the program risks not providing enough oxygen to program hospitals or producing quantities of oxygen too small to justify ongoing maintenance costs.

The program sized plants conservatively, yet large enough to cover hub hospital demand with extra for growth and to distribute to additional health facilities. This sizing kept electricity costs reasonable while providing enough low-cost oxygen to generate cash flow that could cover operations.

However, needs assessments and plant installation were completed in 2018 and 2019, long before the dual crises of the COVID-19 pandemic and the civil conflict in Ethiopia began. Our team greatly underestimated the distance that hospitals would be willing to travel to procure oxygen, the scale of the oxygen need in light of these unprecedented events, and the importance of locally available oxygen sources when transportation lines to the capital city were not available.

Increasing the plant capacity could help relieve some of the oxygen scarcity in the area. Additionally, constructing similar oxygen plants at other strategic locations would also help fulfill demand, reduce cylinder filling times, and potentially further reduce travel time.

Our findings indicate that transportation infrastructure is a critical area for investment to capitalize on the volume advantage of oxygen plants, something that has not been discussed in existing literature. Locating the center of oxygen production comparatively close to target hospitals (within 200 km, compared to 400–500 km previously) was a transformational program success. Hospitals could acquire more oxygen, and some hospitals reported that their cost of oxygen was reduced by 30%–50%, with purchasing data estimates showing an average drop in cost of 56%.

However, last-mile distribution (oxygen delivery to hospital sites and wards) still emerged as a bottleneck. The program had planned to provide last-mile transportation to area hospitals, distributing cylinders directly to hospital sites. Lack of agreement on cost prevented this important step, with the cost of transportation potentially undermining financial sustainability if costs incurred exceeded transportation reimbursement fees. Our findings support the principle that unless last-mile distribution is included, large-volume oxygen programs may result in prohibitively expensive oxygen to some hospitals because of transportation costs. Other hospitals may only be able to afford to procure enough oxygen to meet a fraction of their entire demand. This may exacerbate access disparities and inequity by excluding hospitals outside capital cities entirely or result in these extra costs being passed onto patients.

Our findings support the principle that unless last-mile distribution is included, large volume oxygen programs may result in prohibitively expensive oxygen to some hospitals because of transportation costs.

Insights on the importance of transportation are also relevant for LOX systems, which can be sized to provide even more substantial quantities of oxygen than PSA plants. In many cases, LOX plants are concentrated in capital cities. Piping can be cost-prohibitive, and LOX infrastructure is often missing outside of large tertiary hospitals. Therefore, while a broader catchment of hospitals may be served by a single LOX plant, hospitals procuring oxygen from LOX plants are often required to transport it in cylinders over even longer distances (in the authors’ programmatic experience, this can be hundreds of kilometers in some contexts). Therefore, as interest in the supply power of LOX systems also grows in the context of COVID-19 recovery, learnings on the critical importance of investing in transportation for PSA systems should be extrapolated to LOX implementation as well.

One area of interest for further study is the potential market-shaping impacts of the program. While not queried directly, the program team is aware of new and more localized oxygen distribution hubs that have been put into operation by private companies in direct competition for oxygen sales. There are reports from partners in Ethiopia that spillover effect of increasing market competition has driven costs down regionally and may be indirectly contributing to improved accessibility and affordability of supply.

Altogether, these findings support the adoption of an oxygen ecosystem perspective to improve access to care. The investments made to support transportation, equipment, and clinical training were consistently identified as key enablers of oxygen utilization. However, feedback indicates that these areas would benefit from ongoing investment and that resource bottlenecks have not been completely resolved, putting hospitals at risk in the event of future pandemics that could again lead to dramatic increases in oxygen demand.

### Limitations

Despite the large sample size, this study was limited to Amhara, Ethiopia, and not all results may be generalizable outside this region. However, Amhara shares many characteristics of other low-resource settings struggling to maintain reliable sources of medical oxygen, including electrical supply concerns, lack of oxygen piping, supply chain barriers, and obstacles to regular maintenance. This study is limited to the perspectives of hospital staff at hospitals that procured oxygen from the program PSA plants; hospitals not procuring from the plants may have experienced different bottlenecks. Future research on the perspectives of patients’ experiences receiving oxygen and oxygen plant staff perspectives on plant operations, maintenance, and sustainability, as well as government perspectives on financing, could elicit additional insights and triangulate findings. Lastly, the COVID-19 pandemic and political violence likely drove patterns in supply and demand. Although this study provided a useful snapshot of oxygen access during this period, some of these patterns may be changing in the context of pandemic recovery.

## RECOMMENDATIONS

Based on the results of this qualitative study, we suggest the following recommendations for hospital managers, policymakers, and donors to achieve the maximum impact of investments in oxygen systems.

### Hospital Managers

- Hospital managers should ensure clinical teams are well-trained in both the theory and practical skills of oxygen therapy administration.
- Managers at facilities that regularly pick up medical oxygen should work to estimate oxygen refill timing to ensure sufficient supplies, optimize transport timelines, and plan for spare oxygen cylinders to facilitate the refill process while some patients are still receiving medical oxygen.
- Lack of pressure gauges, regulators, flow meters, pulse oximeters, and single-use consumables like cannulas and masks can pose real barriers, even when a sufficient oxygen supply exists. Ensuring proper sizes and quantities of accessories is imperative to ensure hypoxemic patients receive the treatment they need.

### Policymakers

- When considering high-volume oxygen solutions, policymakers must consider the financial ramifications of their proposed design, particularly for operational expenditures outside the initial capital outlay (plant staff salaries, electricity, maintenance, and spare parts). Some of these operational costs can be covered through low-cost sales to surrounding hospitals. However, clear guidance on how hospitals recoup the cost of the oxygen they provide is needed.
- A well-designed and well-financed distribution plan is imperative for any high-volume oxygen solution serving a general catchment. Throughout much of sub-Saharan Africa, many smaller hospitals and health centers do not have piping, meaning that even oxygen produced through a LOX plant would need to be trucked in cylinders so hospitals can use it. For smaller, remote facilities, transportation costs can meet or exceed the cost of oxygen needed. Ensuring local oxygen production sites, providing oxygen distribution points geographically dispersed throughout a catchment population, or providing a subsidized oxygen delivery service could all reduce oxygen inequity and financial burden to more outlying regions.

### Donors

- Oxygen plants are critical pieces of equipment that have a wide range of health impacts on many critical patient populations. However, the capital expenditure of the plant itself is only a fraction of the total cost of an oxygen plant. Any plant donations must be accompanied by detailed, funded plans on how operational expenditures, backup power, routine maintenance, spare parts, accessories, and distribution will be covered. Procuring or donating plants without addressing these systemic issues is both unwise and irresponsible, as donors risk plant failure that burdens hospitals with expensive equipment that becomes nonoperational far before its estimated lifespan has elapsed. When these issues are addressed, donors can help bring transformational change to a broad spectrum of public health concerns.

## CONCLUSION

The oxygen program’s installation of 2 PSA oxygen plants in Amhara, Ethiopia, brought transformational changes to oxygen accessibility, affordability, and hospital readiness. Pairing PSA plants and ecosystem interventions is an impactful and equitable strategy to close the oxygen gap. Optimizing the medical oxygen system in resource-constrained settings is a critical step toward providing higher-quality patient care to save lives and strengthen health system resilience.

## Notes

**Peer Reviewed**

**First Published Online:** July 18, 2024.
